# Impacts of deforestation on plant-pollinator networks assessed using an agent based model

**DOI:** 10.1371/journal.pone.0209406

**Published:** 2018-12-31

**Authors:** Adrian C. Newton, Danilo Boscolo, Patrícia A. Ferreira, Luciano E. Lopes, Paul Evans

**Affiliations:** 1 Faculty of Science and Technology, Bournemouth University, Fern Barrow, Talbot Campus, Poole, United Kingdom; 2 Biology Department, Faculty of Philosophy, Sciences and Literature of Ribeirão Preto, University of São Paulo, Ribeirão Preto, FFCLRP-USP, São Paulo, Brazil; 3 Departamento de Ciências Ambientais, Centro de Ciências Biológicas e da Saúde, Universidade Federal de São Carlos, São Carlos, São Carlos, Brasil; RMIT University, AUSTRALIA

## Abstract

Plant-pollinator networks have been widely used to understand the ecology of mutualistic interactions between plants and animals. While a number of general patterns have been identified, the mechanisms underlying the structure of plant-pollinator networks are poorly understood. Here we present an agent based model (ABM) that simulates the movement of bees over heterogeneous landscapes and captures pollination events, enabling the influence of landscape pattern on pollination networks to be explored. Using the model, we conducted a series of experiments using virtual landscapes representing a gradient of forest loss and fragmentation. The ABM was able to produce expected trends in network structure, from simulations of interactions between individual plants and pollinators. For example, results indicated an increase in the index of complementary specialization (*H*_*2*_*’*) and a decline in network connectance with increasing forest cover. Furthermore, network nestedness was not associated with the degree of forest cover, but was positively related to forest patch size, further supporting results obtained in the field. This illustrates the potential value of ABMs for exploring the structure and dynamics of plant-pollinator networks, and for understanding the mechanisms that underlie them. We attribute the results obtained primarily to a shift from specialist to generalist pollinators with increasing forest loss, a trend that has been observed in some field situations.

## Introduction

Pollination is a critically important process for the functioning of most terrestrial ecosystems, and animal-pollination is also widely recognised as an ecosystem service of significant value to humanity. Some 75% of food crops and around 90% of wild flowering plants depend at least to some extent on animal pollination [1, 2]. Evidence suggests that pollination services may contribute more than €200 billion annually to the global economy [2, 3], and that global food production is becoming increasingly dependent on animal pollination [4, 5]. At the same time, many pollinators are increasingly under threat from human activities, including climate change, habitat loss and use of insecticides [2, 6]. As a result, a lack of wild pollinators is leading to a widespread yield gap in crop production, which is negatively affecting rural livelihoods [7]. Land management approaches are therefore required that can support and enhance populations of pollinators in agricultural landscapes [2, 7]. Such approaches should be based on an understanding of the ecological factors affecting the dynamics, structure and function of pollinator communities, at both local and landscape scales [8]. Alteration of landscapes by human activity can have major impacts on the distribution of nesting and foraging resources for wild pollinators such as bees, which can potentially affect their ability to pollinate agricultural crops, yet little is currently known about these processes [9,10].

One of the principal approaches to understanding the ecology of pollinator communities involves the analysis of plant-pollinator networks [11]. Such networks are typically constructed by recording the number of pollinator visits to flowers, then by considering both plants and pollinators as network nodes, with pollination interactions forming the links between them. A large number of studies of pollinator networks have now been undertaken, which have provided a variety of insights. For example, many networks have been found to be nested, with the core of the network comprised of highly connected generalist species, and more specialist species interacting with only a subset of these generalist species [12]. A further generalisation is that the number of interactions in such networks tends to increase with network size, following a power-law relationship [12]. However, relatively few studies have been conducted into the impacts of habitat loss and fragmentation on the structure of pollinator networks [9, 13]. For example, Aizen et al. [14] found that mutualistic interactions were lost non-randomly as habitat area declined, as generalist species were more likely to persist than relatively specialist species. Plant-pollinator interactions present in smaller habitat patches tended to be nested subsets of those recorded in larger patches. Similarly, Burkle et al. [15] observed non-random loss of bee species occurring as a result of habitat loss over a period of 120 years, with relatively specialised species being most vulnerable. This was associated with a reduction in redundancy in the network structure and a weakening of interaction strengths [15].

Despite the valuable insights provided by analysis of pollinator networks, their construction faces a number of challenges and limitations. Principal among these is the difficulty of detecting all the interactions that take place between plant and pollinator species, which often requires a substantial investment of time and effort, particularly in species-rich communities [16]. Consequently, detection of species and the interactions between them can often be partial, leading to a potential source of measurement error. Networks are therefore often characterised by an unknown degree of uncertainty, relating to the extent of under-sampling, which can hinder interpretation of the results obtained [16, 17]. The ecological understanding gained from pollinator networks may also often be limited by a lack of information about life history traits and ecological functions of the different species involved [18]. While a number of statistical approaches have been developed to overcome such limitations [19], these can also be demanding in terms of the amount and type of independent data required [16].

As a result of potential sampling error, and the difficulties of achieving a comprehensive sample of plant-pollinator interactions, the mechanisms responsible for variation in the structure and functioning of pollinator networks remain unclear [17]. There is therefore a need to develop spatio-temporally explicit methods that can be used to estimate or eliminate sampling effects, while also enabling the simultaneous evaluation of multiple mechanisms [17]. Potentially, agent based models (ABM) could be of value in this context. Such models are characterised by the explicit representation of individuals as autonomous decision-making ‘agents’, which can interact with each other and their environments. As complex behaviour can arise from very simple ABMs, such models are attracting increasing interest from researchers as a tool for exploring the dynamics of ecological systems [20]. A key advantage of this modelling approach is its ability to capture emergent phenomena of systems from relatively simple sets of rules governing individual behaviour. ABMs are also highly flexible, and can provide a relatively natural description of a system [21].

A number of previous studies have employed ABM approaches to simulate the movement of individual bees and the process of pollination, particularly in honeybees (e.g. see [22–24]). For example, Becher et al. [25] describe a software tool, BEESCOUT, which enables exploration of how bees explore a landscape and distribute their scouting activities over time and space. As part of this software tool, an ABM determines the detection probabilities of food patches by bees, using different search strategies. Outputs from this model can be used as input for the BEEHAVE model of honeybees, which can be used to explore bee colony dynamics in response to different stressors [26]. A further example is provided by Qu et al. [27], who describe EcoSimInGrid, a spatially explicit agent-based model designed to simulate the effects of shared pollination services on plant communities, enabling the relative effects of shared pollination and habitat productivity on community diversity to be analysed. However, we are not aware of any other ABM that has been explicitly designed to simulate the structure of plant—pollinator networks.

Here we describe a spatially explicit ABM of bee foraging and flower visitation, which simulates the movement of individual bees across a heterogeneous landscape, and captures the number of visits made by members of a community of bee species to flowers of different plant species. Model outputs can be used directly to analyse the structure and dynamics of plant-pollinator networks in a similar way to the approaches used for analysing field data. We apply the model to analysis of pollinator networks along gradients of forest loss and fragmentation, using the Atlantic Forests of Brazil as a case study. This region is of global conservation importance owing to the high species richness and endemicity of both plant and insect species, as well as of many other species groups [28]. The area has suffered from intense deforestation in the past, and remaining forest areas are highly fragmented; more than 80% of the fragments are <50 ha, almost half the remaining forest is <100 m from a fragment edge, and the mean distance between fragments is >1.4 km [28]. Evidence suggests that forest fragments are losing species through the disruption of key ecological processes such as pollination and seed dispersal [29, 30]. Recent field-based research in this region has documented a number of relationships between the extent of forest loss and the structure of pollinator networks [31, 32]. Here we examine whether these relationships are robust, such that they can be replicated in a virtual environment where sampling of both plant and bee communities is comprehensive, rather than being the consequence of inadequate sampling in the field. Specifically we use the ABM to test the hypothesis that increasing forest loss and fragmentation reduces network size, increases nestedness and reduces complementary specialization (*H*_*2*_*'*) of pollinator networks, by differentially affecting the relatively specialized interactions that predominate in intact forest [14, 32]. In addition, we hypothesize that the relationships between network characteristics and patterns of forest loss may be non-linear and characterised by threshold responses [33].

## Materials and methods

### Model specification

The model was developed using the Netlogo programming environment [34], a widely used platform for developing ABMs [20] that has the advantage of easily incorporating GIS data. The model was constructed using a world of dimensions 100 x 100 Netlogo grid cells, within which bees can search for food sources. In this study, a grid cell (pixel) was assumed to represent an area of 10 x 10 m, providing an overall landscape of 1 km^2^ in area. Potentially, landscape maps could be imported in a number of different formats, including image files (e.g. BMP, JPG or PNG). Here maps were imported as GIS files in ASCII format, using the Netlogo GIS extension. The maps employed a binary classification of land cover, with categories of ‘forest’ and ‘open’ (non-forest) vegetation. Floral resources were located by randomly selecting individual Netlogo grid cells, with different flower species associated with different grid cell colours, and with ten grid cells of each flower species. A total of 20 flower species were incorporated in the model simulations described here; ten were restricted to forest and ten to open vegetation. The world was set not to wrap either vertically or horizontally.

Bees were created as an additional class of agents, which were able to move across the landscape while foraging for floral resources. A total of 20 bee species were created, represented by different colours. Each species belonged to one of three functional groups, with contrasting behaviour: ‘specialist’, ‘generalist’ and ‘super-generalist’ (see S1 File), with nine, eight and three species in each group, respectively. The nesting location of each species was defined as the starting point of the foraging journeys undertaken by the bee agents. Based on preliminary field observations, nesting locations of specialist species were located randomly within forest vegetation only, whereas nesting locations of generalist and super-generalist species were located randomly across the entire landscape, irrespective of vegetation type.

The model was designed to simulate foraging flights undertaken by bees in search of nectar and pollen resources. Bee movement was simulated as a correlated random walk. Turning angles were randomly drawn from a distribution derived from empirical values obtained for searching bumblebees [25]. At each timestep (tick), the bees moved forward one step. The steplength was drawn randomly from a normal distribution, the mean of which could be specified by the user using a slider on the model interface. For the simulations presented here, a mean step length of 2 was selected, representing 20 m; this choice was based on our preliminary field observations. The total movement distance during foraging flights was determined by the energy levels of each individual bee agent. Initial energy values differed between the bee functional groups, with values of 200, 100 and 50 adopted for super-generalist, generalist and specialist species respectively (see S1 File). Bee functional groups also differed in terms of which flower species could be visited. Whereas specialist bee species could only visit a single flower species, generalist species could visit four, and super-generalists eight. These values were guided by empirical data [31, 32]. For the latter two groups, bee species were allocated randomly to individual plant species (see S1 File).

The amount of energy used during the bee flight was proportional to the distance moved at each timestep, with a single unit representing the amount of energy required to move a distance of one cell (10 m). As a result, total flight length was higher for super-generalist than for generalist species, and higher for generalist than specialist species, reflecting field observations. Once the energy had been used, the foraging flight was terminated; the return flight to the hive was not simulated since our intention was to focus on recording bee-plant interactions.

The model was designed to capture flower visitation events, which took place when a bee entered any grid cell that was defined as an appropriate flower for the focal bee species according to bee profiles (Table C in S1 File). In nature, bees will detect floral resources through a combination of visual and olfactory cues, but it is unclear at what range such cues have an effect, or how this varies among bee species. The model therefore assumed no detection by the bee agents until the bee was coincident with the flower grid cell, following Becher et al. [25]. The number of visits of each bee species to each flower species was summed for all of the individuals present in each simulation. The presence of different flower species varied between the different experiments conducted (see Tables A and B in S1 File), but the overall density of both flowers and bees remained constant for all simulations (10 individuals of each bee species and 20 individuals of each flower species). A bee could potentially visit more than one flower on an individual foraging flight. Code for the model is provided in S2 File.

### Model experiments

Model experiments were conducted to evaluate the impact of forest loss and fragmentation on network structure by creating gradients of deforestation. Eleven different values of forest cover were examined, namely 0%, 10%, 20%, 30%, 40%, 50%, 60%, 70%, 80%, 90% and 100%. Each deforestation gradient was replicated ten times, producing 110 unique input maps in total. All simulations were continued until foraging flights of all bees were complete (150 ticks).

The software program GradientLand [35] was used to generate land cover maps, which were imported into Netlogo to conduct the experiments. GradientLand is designed to simulate sets of habitat loss gradients as random and fractal neutral landscapes. It differs from other landscape generator programs by producing a sequential gradient over the same original landscape, which is designed to mimic the sequential habitat removal processes observed in the real world. Patches of a particular habitat type can only lose habitat from patch edges, and as a result the overall patch shape and fractal pattern are preserved. GradientLand produces landscapes with relatively stable pixel aggregation and patch shapes along gradients of habitat loss, especially for landscapes with highly clumped habitat patches (high values of H, the Hurst exponent used to generate base fractal surfaces [35]). Here an H value of 0.9 was used in all simulations, which is typical of current landscapes in the Atlantic Forest region of Brazil (D. Boscolo, unpublished data). The default value of five was used as a random seed. Sensitivity analysis was conducted by repeating model runs with different allocation of bee to flower species (see Table D and Fig B in S1 File).

### Data analysis

The maps generated by GradientLand were analysed using FRAGSTATS v.4 [36] to characterise landscape pattern. We used four class metrics as generated by FRAGSTATS, following Ferreira et al. [31, 32]: (1) Forest Cover (PLAND), calculated as the percentage of forest cover in the landscape; (2) Landscape Connectance Index (CONNECT), which represents the percentage of connections between forest patches less than 50 m apart (considered as a functional connection based on bees’ foraging flight capabilities), relative to the maximum possible number of connections among all patches of a given landscape; (3) Mean Patch Area (AREA_AM), referring to the mean area of all forest patches within the 1 km^2^ study area; and (4) Mean Patch Shape Index (SHAPE_AM), calculated as forest patch perimeter (m) divided by the square root of forest patch area (m^2^), adjusted by a constant [36]. Owing to the presence of very small patches within the landscapes, we also calculated AREA_MN and SHAPE_MN weighted by the proportional contribution of each forest patch to the total area of remaining forest in the landscape. This approach reflects the fact that larger habitat patches could potentially have a greater influence on the structure of pollinator networks than smaller ones [31].

In each experiment, we calculated a number of measures describing the structure of plant-bee networks: (i) connectance, namely the proportion of possible links in the network, calculated as the sum of links divided by the number of cells in the matrix of plant and bee species; (ii) nestedness, namely the extent to which specialist species interact with specific subsets of generalist species and with themselves; (iii) *H*_*2*_*'*, an index of complementary specialization, representing the extent of reciprocal specialist interactions; values range between 0 and 1, lower values corresponding to networks with less specialized interactions; (iv) network size, namely the sum of the total number of bee and plant species in the network; (v) asymmetry of the network, positive values indicating more plant species and negative values indicating more bee species. All metrics were calculated using the bipartite package in software R [37].

To assess how landscape affected plant-bee interaction network structure we used Generalized Linear Models, with a Poisson error distribution. To assess whether any explanatory variables were correlated with each other, we applied Spearman rank correlation tests. All analyses were performed using the software R [37].

## Results

**Fig 1:**
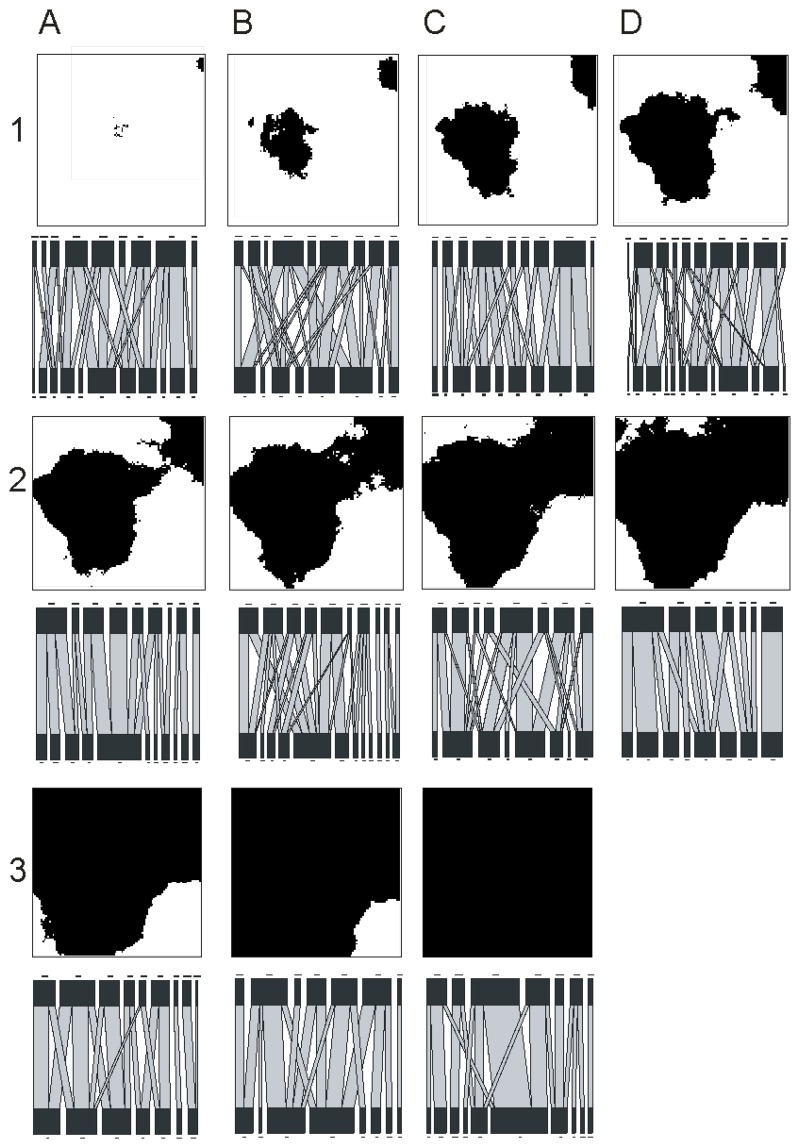
Example of forest cover gradient, generated using GradientLand, and associated pollinator networks produced from model output (for details, see text). Forest cover, illustrated in black: A1, 0%; B1, 10%; C1, 20%; D1, 30%; A2, 40%; B2, 50%; C2, 60%; D2, 70%; A3, 80%; B3, 90%; C3, 100%. In the network diagrams, plant species are illustrated on the higher row, and bee species on the lower row. The widths of the connecting lines are proportional to interaction strength. The rectangles represent species, and the width is proportional to the sum of interactions involving this species.

Analysis of the virtual landscapes using FRAGSTATS indicated that the percentage forest cover values differed slightly from the descriptors of the experimental treatments (Table 1). For example, for the 0% cover treatment, the actual mean value of the ten replicates obtained in the virtual landscapes was 0.79%, indicating the presence of some forest (Fig 1). This reflects the stochastic element incorporated in GradientLand when generating fractal landscapes, arising from use of a random seed. Mean patch size of forest increased exponentially with an increase in forest cover, whereas area weighted mean patch size increased linearly (Table 1). Mean patch shape index declined gradually with increasing forest cover, but when weighted by area, shape index values displayed a different response, reaching a maximum value at 50% forest cover. Landscape connectance did not show a consistent response with forest cover, values often differing markedly between one forest cover category and the next, along the deforestation gradient (Table 1). In general, in landscapes with relatively low forest cover, most forest was located within a small number of relatively large patches, rather than being divided into a large number of small fragments (Fig 1). This reflects the high fractal index (0.9) used to generate the landscapes and also the forest distribution expected for the Atlantic Forest region [28].

**Table 1:** Characteristics of virtual landscapes used in the model experiments, generated by GradientLand.

ET	PLAND (%)	AREA_MN (ha)	AREA_AM (ha)	SHAPE_MN	SHAPE_AM	CONNECT
0	0.79 ±0.03	0.38 ±0.10	0.56 ±0.08	1.40 ±0.09	1.60 ±0.08	29.1 ±9.53
10	10.40 ±0.11	2.76 ±0.50	8.77 ±0.51	1.40 ±0.05	2.00 ±0.10	45.52 ±11.29
20	20.37 ±0.15	4.73 ±1.78	16.37 ±1.07	1.41 ±0.08	2.06 ±0.12	22.43 ±6.01
30	30.42 ±0.15	5.49 ±1.39	25.18 ±1.61	1.44 ±0.07	2.35 ±0.21	33.58 ±9.51
40	40.43 ±0.16	7.68 ±3.64	33.49 ±2.68	1.35 ±0.02	2.06 ±0.16	15.33 ±2.59
50	50.53 ±0.14	11.50 ±4.44	44.25 ±2.87	1.39 ±0.05	2.28 ±0.13	22.0 ±4.74
60	60.57 ±0.13	13.94 ±5.29	54.66 ±3.47	1.33 ±0.04	2.18 ±0.13	21.74 ±4.01
70	70.65 ±0.14	17.55 ±2.54	69.71±0.52	1.39 ±0.07	2.17 ±0.19	50.0 ±8.01
80	80.80 ±0.13	25.62 ±7.10	80.31±0.36	1.26 ±0.03	1.83 ±0.13	33.74 ±9.45
90	90.90 ±0.09	38.16 ±9.72	90.61±0.18	1.26 ±0.04	1.60 ±0.10	52.43 ±12.01
100	99.99 ±0.00	99.99 ±0.00	99.99 ±0.00	1.01 ±0.00	1.01 ±0.00	0 ±0.00

Values derived from FRAGSTATS (see text). Values presented are means (n = 10) ± SE. Abbreviations: ET, experimental treatment; PLAND, percentage of landscape covered by forest; AREA_MN, mean patch size; AREA_AM, area weighted mean patch size; SHAPE_MN, mean patch shape index; SHAPE_AM, area-weighted mean patch shape index; CONNECT, connectance index. Details of the metrics are given by McGarigal et al. (2012).

**Fig 2:**
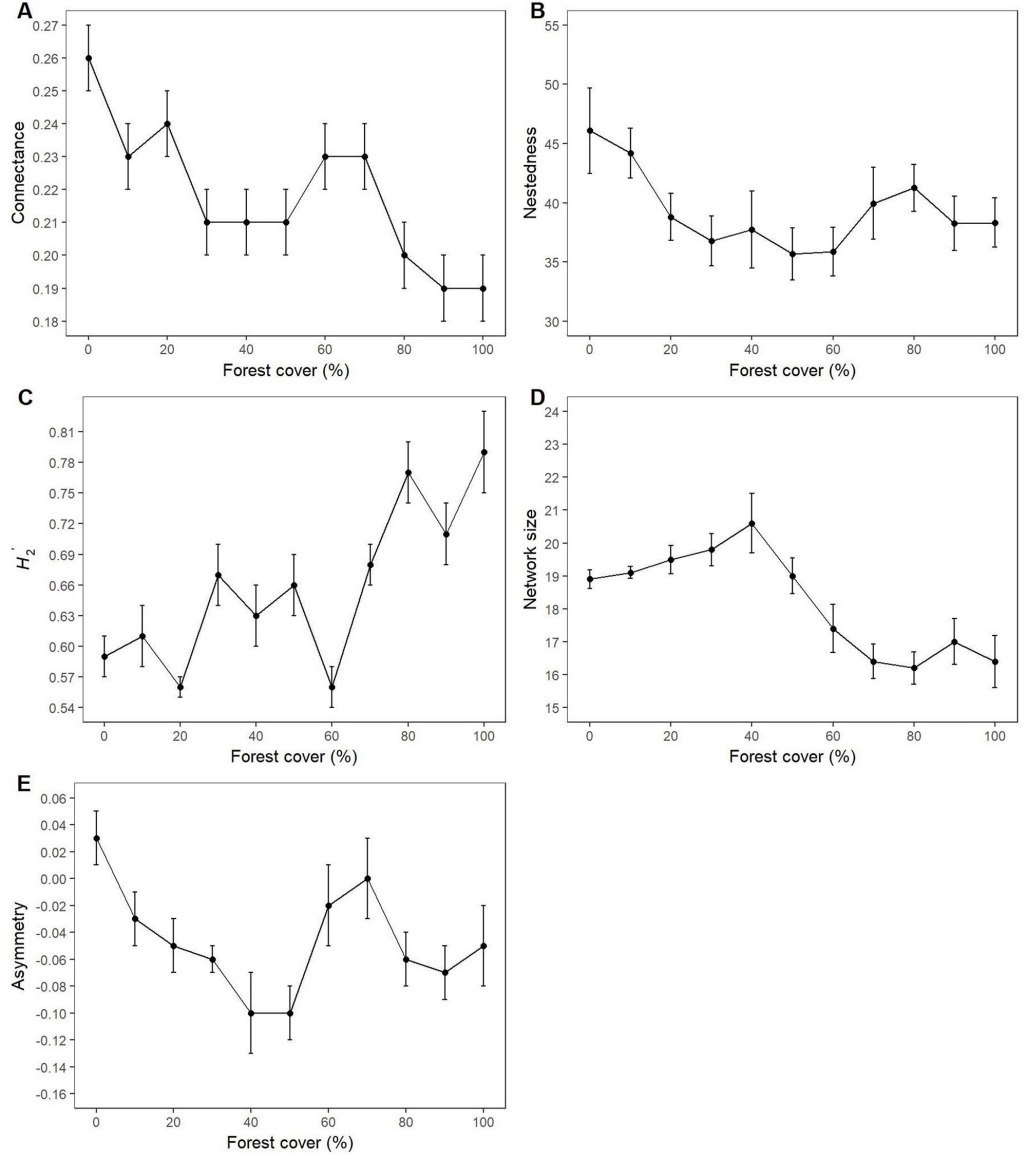
Relationship between forest cover and the structure of pollinator networks derived from model output. Values presented are means ± SE (*n* = 10). A. Connectance, the realised proportion of possible links. B. Nestedness, the extent to which specialist species interact with specific subsets of generalist species. C. H_2_’, a measure of network specialisation. D. Network size, the number of plant and bee species per network. For details of calculation, see text.

Visual assessment of network diagrams tended to indicate a general decrease in network connectivity, and an increase in specialisation, with increasing forest cover (Fig 1). When quantitative measures were analysed, significant variation was observed in each of the four measures of network structure across the deforestation gradients (Figs 1 and 2). However, in no case was this variation systematic or characterised by a simple overall linear relationship. There was a general tendency for network connectance and nestedness to decline with increasing forest cover, until values of around 50% cover were reached; trends above this threshold value were less apparent. Conversely, *H*_*2*_*'* and network size tended to increase with increasing forest cover, again until cover values of around 50% were reached. Thereafter, network size tended to decline, whereas the highest values of *H*_*2*_*'* were associated with forest cover values of >70% (Fig 2).

Regression analysis revealed a number of relationships between the structure of pollinator networks and the spatial pattern of virtual landscapes (Table 2). Negative relationships were recorded between network connectance and percentage of forest cover, and both weighted and unweighted mean patch size. Similar results were obtained for network size. In contrast, *H*_*2*_*'* was positively related to percentage of forest cover, and both weighted and unweighted mean patch size, but was negatively related to both weighted and unweighted mean patch shape. Network nestedness was positively related to mean patch shape and connectance, but not to the other landscape metrics, whereas network asymmetry was not related to any of the landscape metrics (Table 2).

**Table 2:** Relationships between the structure of simulated pollinator networks and the spatial pattern of virtual landscapes along a gradient of forest loss, determined using generalized linear models. Landscape pattern metrics were generated using FRAGSTATS (see Table 1).

	Estimate	Std. Error	t value	Pr(>|t|)
Connectance				
PLAND	-0.000516564	9.86E-05	-5.238529001	<0.001
AREA_MN	-0.000425145	0.000107713	-3.94703102	<0.001
AREA_AM	-0.000499433	9.47E-05	-5.275108581	<0.001
SHAPE_MN	0.027680759	0.016801954	1.647472648	0.102
SHAPE_AM	0.006618329	0.006303062	1.050018101	0.296
CONNECT	6.36E-05	0.000124251	0.5120134	0.610
Nestedness	Estimate	Std. Error	t value	Pr(>|t|)
PLAND	-0.044391605	0.025480796	-1.742159256	0.084
AREA_MN	-0.006705688	0.026949709	-0.248822295	0.804
AREA_AM	-0.035382611	0.024607112	-1.437901801	0.153
SHAPE_MN	9.859955389	3.865428319	2.55080539	0.012
SHAPE_AM	-1.516447611	1.475006035	-1.028095869	0.306
CONNECT	0.06276676	0.028472054	2.204504115	0.030
H_2_'	Estimate	Std. Error	t value	Pr(>|t|)
PLAND	0.001793754	0.000290499	6.174728071	<0.001
AREA_MN	0.001878867	0.000302687	6.207290541	<0.001
AREA_AM	0.001841068	0.000272348	6.759973344	<0.001
SHAPE_MN	-0.162683152	0.049646195	-3.276850388	0.001
SHAPE_AM	-0.061742598	0.018452599	-3.34601093	0.001
CONNECT	1.41E-05	0.000380664	0.036921512	0.971
Network size	Estimate	Std. Error	z value	Pr(>|z|)
PLAND	-0.002040696	0.000707432	-2.88465278	0.004
AREA_MN	-0.001563505	0.000771097	-2.027637237	0.043
AREA_AM	-0.002029303	0.00068357	-2.968681279	0.003
SHAPE_MN	0.18303377	0.106282999	1.722135915	0.085
SHAPE_AM	0.06188097	0.040272089	1.53657215	0.124
CONNECT	-0.00017342	0.000798296	-0.217237312	0.828
Asymmetry	Estimate	Std. Error	t value	Pr(>|t|)
PLAND	-0.000368197	0.000240108	-1.533463182	0.128
AREA_MN	-3.84E-05	0.000253213	-0.151547564	0.880
AREA_AM	-0.000210453	0.000232482	-0.905243962	0.367
SHAPE_MN	0.012823678	0.037369521	0.343158751	0.732
SHAPE_AM	-0.007374806	0.013905834	-0.530339011	0.597
CONNECT	0.000236462	0.000272471	0.867840627	0.387

Abbreviations: PLAND, percentage of landscape covered by forest; AREA_MN, mean patch size; AREA_AM, area weighted mean patch size; SHAPE_MN, mean patch shape index; SHAPE_AM, area-weighted mean patch shape index; CONNECT, connectance index. Error distributions were *Gaussian* with the exception of network size, which was *Poisson*. For details of network structure measures, see text.

The generalised linear model was conducted using a single model for each individual factor. Interpretation of these results should be based on consideration of correlations between these factors. Correlation analysis identified significant correlations between the percentage of forest cover and all other landscape metrics with the exception of the landscape connectance index. Similar results were obtained with both weighted and unweighted mean patch size. The only variables that were significantly correlated with landscape connectance were both weighted and unweighted shape indices (Table 3).

**Table 3:** Spearman correlation analysis of the relationships among landscape pattern metrics for virtual landscapes along a gradient of forest loss.

	AREA_MN	AREA_AM	SHAPE_AM	SHAPE_MN	CONNECT
PLAND	0.87 *<0*.*001*	0.98 *<0*.*001*	-0.43 *<0*.*001*	-0.27 *0*.*004*	-0.10 *0*.*306*
AREA_MN		0.90 *<0*.*001*	-0.23 *0*.*0171*	-0.34 *<0*.*001*	-0.06 *0*.*544*
AREA_AM			-0.41 *<0*.*001*	-0.25 *0*.*009*	-0.07 *0*.*448*
SHAPE_AM				0.48 *<0*.*001*	0.24 *0*.*011*
SHAPE_MN					0.31 *0*.*001*

Values were derived using FRAGSTATS (see Table 1). Values of correlation coefficient (r) are presented, along with P values (in italics). N = 110. Abbreviations: PLAND, percentage of landscape covered by forest; AREA_MN, mean patch size; AREA_AM, area weighted mean patch size; SHAPE_MN, mean patch shape index; SHAPE_AM, area-weighted mean patch shape index; CONNECT, connectance index.

## Discussion

Results of the landscape pattern analysis were consistent with expectations, and were broadly consistent with those of other studies. It is well established, for example, that different landscape pattern metrics are often highly correlated [38], as observed here. In particular, the percentage forest cover in the simulated landscapes was very closely related to patch area. The FRAGSTATS shape index (SHAPE) measures the complexity of patch shape compared to a square standard shape of the same area [36]. The negative relationship between forest cover and SHAPE observed here is therefore understandable as the patches became increasingly irregular in shape with increasing forest loss, reflected in the increasing values of the index. The landscape connectance index relates to the distance between adjacent patches, and therefore was understandably related to SHAPE. Similar relationships have been identified in real world situations [38]; for example, Ferreira et al. [32] found percentage forest cover and patch area to be highly correlated along gradients of forest loss in the Atlantic Forest of Brazil. Similarly, in an assessment of deforestation in Ecuador, shape index increased as deforestation progressed, and both forest area and mean patch size were again highly correlated [39]. This consistency with field observations supports the use of maps generated by GradientLand in the ABM presented here.

The structure of a pollinator network can be considered as an emergent property of a mutualistic plant-pollinator system. The current results (Fig 2) demonstrate that the ABM was able to produce expected trends in network structure by simulating the interactions between individual plants and pollinators. This illustrates the potential value of ABMs for exploring the structure and dynamics of pollinator networks, and their underlying mechanisms. Specifically, the increase in the index of complementary specialization *H*_*2*_*'* with increasing forest cover is consistent with field observations. *H*_*2*_*'* specifies the degree of specialization in a network, with lower values corresponding to those networks with fewer reciprocal specialist interactions [40]. Specifically, *H*_*2*_*'* quantifies the deviation of observed interactions from those expected given the species’ abundances or interaction frequencies, values ranging from 0 for the most generalized networks to 1 for networks that are completely specialised [41]. A number of studies have reported increasing generalization of plant-pollinator networks with increasing habitat loss. For example, Aizen et al. [14] recorded increasing loss of specialist plant-pollinator interactions with increasing habitat loss in Argentinian sierras, and Fereira et al. [32] reported a similar trend in the Atlantic Forests of Brazil along a gradient of forest cover. Comparable results were obtained by Geslin et al. [42] along an urbanisation gradient in France. Such results are also consistent with theoretical expectations, as plants with specialist pollinators are more vulnerable to disruption of pollination mutualisms as a result of habitat loss, as they are less able to compensate for the loss of their mutualistic partners through forming relationships with other alternative pollinators [43]. Nonetheless, it should be noted that some field evidence contradicts this pattern. For example in a coastal dune marshland community, Traveset et al. [44] found increased specialisation of plant-pollinator interactions following habitat loss. Here, pollinators included beetles, flies and ants as well as bees, highlighting the fact that not all functional groups of pollinators respond similarly to habitat loss.

A second key result obtained from the modelling results presented here was the significant positive relationship observed between network nestedness and habitat patch size, but the lack of any relationship between nestedness and the degree of forest cover. This closely parallels results obtained in field surveys in the understory of Brazilian Atlantic Forests, where Ferreira et al. [32] similarly found that nestedness was positively related to patch size and shape but was not associated with forest cover. Patterns of nestedness in mutualistic networks are the subject of theoretical debate [12, 45, 46], and the response of nestedness to changing land cover is poorly understood. Very few previous studies have assessed how nestedness of plant-pollinator networks changes over a gradient of habitat loss and fragmentation [46]. Spiesman and Inouye [47] note that habitat loss and fragmentation may cause mutualistic networks to disassemble through a process whereby specialist species are lost from a network before more generalist species. In this case, habitat loss and / or fragmentation should lead to a reduction in nestedness because of a decline in the number of interactions between specialists; those species that remain will form a relatively well-connected network of generalists. This provides a potential mechanism for the results observed here and by Ferreira et al. [32], although it is interesting to note that this process was related in both studies to the degree of fragmentation but not of habitat loss. In their research on plant-pollinator networks in pine-oak savannah in Florida, USA, Spiesman and Inouye [47] similarly found no effect of the degree of habitat loss on nestedness; the effects of fragmentation were not explicitly examined in that study.

The negative relationship recorded here between forest cover and network connectance (Table 2) is also consistent with field evidence. Connectance provides an indication of the proportion of all possible interactions in a network that are actually realized [47]. Spiesman and Inouye [47] found a similar negative relationship between habitat amount and network connectance in their research on pine-oak savannah, which they attributed to variation in the number and abundance of pollinator species. Similarly, in our study we attribute this result to the loss of specialist pollinators with increasing forest loss, a pattern that has been observed in the field along deforestation gradients in the Atlantic forests of Brazil [31] as well as in other habitats [14]. It has been suggested that such changes in the structure of plant-pollinator networks could have consequences for their dynamics and viability; both nestedness and connectance may enhance community stability by allowing competitors to facilitate one another by sharing mutualistic partners, thereby reducing the negative effects interspecific competition [47, 48]. Loss of both network connectance and nestedness as a result of habitat loss and fragmentation could therefore reduce the stability of plant-pollinator networks in Atlantic Forest, and potentially undermine their resilience to further environmental change.

In contrast, the negative relationship recorded here between forest cover and network size differs from the results obtained by Ferreira et al. [32] in the Atlantic Forests of Brazil, where the converse trend was observed. It is important to note that the simulated networks presented here differ from these field situations, as they incorporated pollination interactions across the entire landscape (i.e. including both forest and non-forest vegetation), rather than being limited to a single environment type, as was the case in the field studies. This highlights the need for field data for pollinator networks to be collected from both inside and outside forest patches along deforestation gradients; such data are currently lacking. A further difference is that the species richness and abundance of both bees and flower species was constant across the modelled gradient of forest cover, which contrasts with the field situation. In field studies, as habitat area increased, networks tended to become larger and more diverse [14, 32]. The variation in network size encountered in the model results is attributable to stochastic processes, such as chance interactions between bees and flowers arising from pollinator behaviour. As evidence of this, it is notable that in none of the model experiments conducted did all possible interactions between bee and flower species actually occur. It should also be noted, however, that some field studies have reported results that were consistent with the model outputs presented here. For example, working in temperate woodlands, Vanbergen et al. [49] found that plant-pollinator networks from relatively disturbed sites were less connected, but were also more speciose and therefore larger. This was attributed to the effects of disturbance on the size and distribution of interspecific interactions in the networks, and the influence of these factors on robustness to co-extinction cascades. Similar mechanisms were identified by Grass et al. [50] in calcareous grassland fragments, where plant–pollinator communities were found to respond to the loss of species associated with habitat fragmentation by opportunistic partner switches. While such processes have not yet been documented in Atlantic Forest, these results suggest they might usefully be examined in future field investigations.

Although a number of relationships were detected between landscape pattern and the structure of pollinator networks, regression analyses did not fully explain the variation in the model outputs. Many of the measures of network structure varied non-linearly along the forest cover gradient, with contrasting patterns of response either below or above 50% forest cover. Network size, for example, reached a peak around 40% forest cover. This finding is relevant to the concept of landscape-scale thresholds in habitat fragmentation and loss, which have attracted a great deal of attention from both empirical and theoretical researchers, including those working in Atlantic Forest of Brazil [51, 52]. Both modelling and empirical studies have suggested that extinction thresholds can often be observed below 30% of remaining habitat, associated with an exponential increase in the distance among patches at 10–20% of remaining habitat [52]. However, the occurrence of such thresholds is still the focus of debate and controversy, reflecting the fact that species often respond individually to habitat loss [52]. The current research suggests that fragmentation thresholds may also occur in the structure of plant-pollinator networks as a result of species-level responses to deforestation, a novel suggestion that merits further investigation. Such responses may again be attributable to shifting contribution of specialist *versus* generalist pollinator species across the deforestation gradient. However, these features of the results must also reflect the stochastic processes included in the model, including the random distribution of both bee and plant species, and the random elements of bee movement. Such processes are also influential in a field situation, but here the problem of uncertainty is compounded by the additional challenge of achieving adequate sampling. As noted by Vázquez et al. [53], field sampling of pollinator networks is subject to a number of potential observation errors, and by the relative abundance of the species in the network, which influences the probability of observing a particular interaction. The characteristics of mutualistic networks have repeatedly been shown to be influenced by the degree of sampling effort. For example, the number of species and links within a network tend to increase with the number of observations made. Evidence suggests that nestedness, however, may be relatively insensitive to sampling effort [19, 54]. Potentially, the model described here could be used to explore such sampling issues, for example by evaluating how network metrics vary with the abundance of both bees and flowers, and the duration of model runs, which is effectively a proxy for sampling effort.

While the model as presented here was assessed in relation to a particular ecosystem, namely the Atlantic Forests of Brazil, it could readily be adapted to a wide range of other ecological situations. The GIS extension of Netlogo enables real-world maps of landcover to be imported into the model, which could potentially be integrated with field data describing the distribution of different plant and pollinator species. The model could be further developed in a number of additional ways. Here, the model was configured such that nine bee species only visited a single plant species, whereas only two plant species were limited to a single bee species. This is consistent with the fact that plant-pollinator relationships have often been observed to be highly asymmetric and nested, whereby species with relatively few partners primarily interact with subsets of a generalized core group of partners [12, 17, 53, 55]. However, the numbers of specialist and generalist bee species could readily be changed to represent communities with different characteristics. While three different functional types of bees were included here, this could be extended to include additional morphological and behavioural variation among pollinators, as well as variation in life history traits of plant species. This could enable the functional importance of network structure to be evaluated. Recent research conducted in the Seychelles has provided evidence that pollinator network structure can relate to pollination efficiency and fruit set [56], processes that could also potentially be simulated. The model as presented here was limited in its simple binary classification of land cover; much more elaborate vegetation maps could potentially be incorporated. This would enable the role of landscape heterogeneity to be explored, a factor that has previously been shown to have a major influence on the structure of plant-pollinator networks in tropical savannah ecosystems in Bahia, Brazil [57] and in the Atlantic Forest [58]. The model could also be used to examine the resilience of pollinator networks, for example by simulating the removal or extinction of individual species, and observing the impacts on network structure (c.f. [59]).

The principal value of this modelling approach is that it provides a tool to examine the mechanisms underlying the structure of pollinator networks in a realistic way. A variety of different mechanisms have been proposed as an explanation of patterns in network structure, including neutrality, trait matching among interacting species, phylogenetic constraints and sampling artefacts [17, 60, 61]. However, relatively little is known about the relative importance of these mechanisms, and very few studies have attempted to evaluate multiple mechanisms simultaneously, partly because of the methodological challenges involved [17]. Modelling approaches such as the ABM presented here could make a valuable contribution in this area, by enabling the relationship between network structure and the relative influence of different mechanisms to be explored. This could be achieved by conducting experiments *in silico*, as described here in relation to forest fragmentation. A similar issue relates to the use of metrics to describe landscape pattern. Although a large number of metrics have been developed, their relationships with ecological processes have rarely been tested, and as a result their practical value has been questioned [62]. Again, the relationship between landscape pattern and pollinator behaviour, and ultimately pollinator network structure, can be explored using this model, as demonstrated here. In this study, relationships between landscape pattern and network structure emerged even though pollinator movement was essentially random; no explicit process linking pollinator behaviour to landscape pattern was included in the model.

## Supporting information

S1 File Appendix. Details of study area and model.
(DOC)

S2 File Netlogo model employed in the research. (ZIP)

S3 File Model description according to the ODD protocol. (DOC)
