# Supplementary material for: Impacts of deforestation on plant-pollinator networks assessed using an agent based model
Source: PLoS One. 2018 Dec 31;13(12):e0209406. doi: 10.1371/journal.pone.0209406 (PMC6312366; doi:10.1371/journal.pone.0209406)
Supplement: S1 File — Details of study area and model. (DOC) [file pone.0209406.s001.doc]

**S1 Appendix. Details of study area and model.**

*Study area and application to model*

Pollination in the Atlantic Forest of Brazil is undertaken by a diverse community of bees, wasps, beetles, flies, and other invertebrates, as well as birds and bats. Here, we focus explicitly only on bees. The bee community is characterised by a high diversity of species, many of which are poorly understood ecologically, and some of which are poorly defined taxonomically. The bee community comprises both social and solitary bee species, as well as some eusocial species. Field observations suggest that while the majority of bee species are social, a substantial minority are solitary (Michener 2007). Observations also suggest that species differ in their degree of association with forest cover both for nesting and foraging (Steffan-Dewenter and Tscharntke 1999, Brosi et al. 2008).

Originally, the land cover of this region was largely covered in forest, so the current bee community must essentially be derived from one that was originally associated with forest. However, as a result of a long history of human intervention in the region, the bee community has been significantly modified from what was present originally (Brosi et al. 2008). Based on preliminary field observations, bee species appear to differ in their dependence on forest for nesting sites (eg. in standing or fallen trees), with social bees particularly dependent on forest habitat. The nesting requirement of different species was not explicitly included in the model described here, except for the fact that some forest-dependent species were located within forest habitat at the onset of model runs, whereas others were located randomly across the entire landscape. In this way, the model simulates foraging journeys made by bees from their nest location, but does not explicitly incorporate return journeys to the nest.

Field observations in this region suggest that social bee abundance can increase with a decline in forest cover (Ferreira et al. 2015), but this was not explicitly modelled here. Rather, constant abundances of both plant and bee species were modelled across the deforestation gradient, to control for these factors. However the model did differentiate between plant species situated within and outside forest areas. The existence of floral resources outside forest areas depends critically on the type of land use surrounding forest patches, which in this region is mostly agricultural or urban land (Boscolo et al. 2017). Some types of agriculture, such as the sugar cane cultivation that is widespread in the region, would likely be associated with low flower densities. Pasture areas could potentially contain higher flower densities than forest, depending on the intensity of grazing. Here we specified ten flower species that were restricted to forest habitat, and ten that were restricted to open habitat, with flower species richness varying along the deforestation gradient, as specified in Table A. Details of the flower species included in the model are given in Table B. We employed values of flower diversity that are generally comparable with values recorded in the field (Ferreira et al. 2015, Boscolo et al. 2017).

Although there may be bee species that are obligately associated with non-forest areas, we do not have evidence for such species at the current time in this region. However field observations suggest that there are bee species that are only encountered in forest areas, and are limited to forest for nesting and / or foraging. On the basis of field observations, we therefore identified three main groups of bee species: (i) specialists, which are dependent on forest for nesting, and only forage over relatively short distances; (ii) generalists, which display greater flexibility in terms of the number of flower species that they can pollinate and in terms of their nesting requirements; they forage over intermediate distances; (iii) supergeneralists (Giannini et al. 2015), which visit a wider range of flower species, are flexible in terms of their nesting location, and tend to forage over wider areas than the other groups. The bee types included in the model are presented on Table C. However we emphasize that information is lacking on the biology of most bee species in this region. We have attempted here to identify these major groups so that the outputs generated by the model might be comparable to a field situation to enable model testing. In the model simulations presented here, we included nine specialist species, eight generalists and three supergeneralist species. These values were based on our preliminary field observations relating to the composition of the bee community in the Atlantic Forest region (Ferreira et al. 2015, 2018; Boscolo et al. 2017). Clearly, if different numbers of generalist and specialist bee species had been included in the simulations, different results would likely have been obtained.

*Modelling bee movement*

Bee movement distances have not been accurately measured in the Atlantic Forest region. We recognise that there are some groups of bee species, such as euglossine bees, which can travel very long distances when dispersing (Ramalho et al. 2009). These were not explicitly considered by the model as currently configured.

There is a debate in the literature regarding whether or not bees demonstrate Lévy flight patterns when foraging. Here we follow Reynolds (2009) who found that Lévy flight patterns in foraging bumblebees are rare, and are not part of an innate, evolved optimal searching strategy. For this reason, we did not include Lévy flight patterns in the model simulations described here, although as currently configured, bee movement according to a Lévy flight is provided as an option in the model code.

Bee movement was simulated here as a correlated random walk, which has been widely found to be effective in describing movement patterns of a range of animal species, including bees (Lenz et al. 2013). Other studies that have modelled bee movement in this way include Marchand et al. (2015) and Everaars and Dormann (2014). Natural variability is a major element of the correlated random walk. For this reason, step length was specified as a variable in the model, which can be altered by the user through a slider on the main model panel. The step length was drawn from a normal distribution with the mean value specified by the slider. The typical step length of foraging bees in the Atlantic Forest region is not accurately known, but we selected a mean value of 20 m (i.e. two Netlogo grid cells each of 10 m) for the simulations presented here, based on our field observations. The turn angles were derived from empirical data of bumblebee movement derived from harmonic radar, as specified in the main text, sourced from Becher et al. (2016). The model code for bee movement was adapted from that provided by O’Sullivan and Perry (2013).

In the model, the landscape is a square kilometer in size and consists of many patches of forest in a non-forest matrix. Bees were able to forage throughout the landscape, but were only able to visit selected flower species as specified in Table C. Flower species were distributed randomly in either forest or non-forest habitat, depending on the species involved, as specified in Table B. The bees differ in maximum foraging distance and number of plant species they can pollinate, as specified in Table C. The model simulates a single foraging journey undertaken from the nest location, which was distributed randomly in either forest or non-forest habitat, depending on the species (Table C). Bee numbers were constant throughout all model simulations (20 individuals per species). Composition of the flower community varied along the deforestation gradient (Table A) such that the overall richness and abundance of plant species was constant (i.e. 10 plant species with 20 individuals per species). Therefore the species richness of both plant and bee species, plus overall abundance, were constant across the deforestation gradient; the only variable that changed was the composition of the plant community.

It should be noted that the model was configured to permit only a single type of flower species to occur within a single Netlogo grid cell (pixel). Given that each cell was assumed to represent an area of 10 x 10 m, this obviously represents a simplification, as in the field situation, it would potentially be possible for multiple flower species to co-occur at that scale. However, the values employed here were based on field observations conducted in the Atlantic Forest of Brazil, where floral resources used by bees typically occur at low densities in the forest understorey, and a single floral resource within an area of 10 x 10 m is not unrealistic. The overall density of floral resources was kept constant across the deforestation gradients (Table A) as the focus here was on examining the effect of forest fragmentation on plant-pollinator network structure, rather than the effect of variation in flower availability.

*Plant species characteristics*

All individual plants were located randomly in either forest or non-forest (open) habitat, as detailed on Table A.

Table A. Distribution of the plant species along the deforestation gradient.

| Extent of forest cover (%) | No. of forest habitat plant species | No. of open habitat plant species | Plant species identification numbers present |
| --- | --- | --- | --- |
| 100 | 10 | 0 | 1-10 |
| 90 | 9 | 1 | 1-9, 11 |
| 80 | 8 | 2 | 1-8, 11-12 |
| 70 | 7 | 3 | 1-7, 11-13 |
| 60 | 6 | 4 | 1-6, 11-14 |
| 50 | 5 | 5 | 1-5, 11-15 |
| 40 | 4 | 6 | 1-4, 11-16 |
| 30 | 3 | 7 | 1-3, 11-17 |
| 20 | 2 | 8 | 1-2, 11-18 |
| 10 | 1 | 9 | 1, 11-19 |
| 0 | 0 | 10 | 11-20 |

Table B. Characteristics of plant species included in the model. The flower colours represent the Netlogo colour descriptors or numerical codes. *The number of bee species able to visit each plant species was assigned randomly; see Table C. O and F refer to Open and Forest habitat, respectively.

| Plant species identification number | Flower colour (Netlogo code) | Habitat | Total number of visiting bee species* |
| --- | --- | --- | --- |
| 1 | Black | F | 1 |
| 2 | Red | F | 4 |
| 3 | Pink | F | 3 |
| 4 | Orange | F | 3 |
| 5 | Brown | F | 3 |
| 6 | Magenta | F | 1 |
| 7 | 18 | F | 2 |
| 8 | 28 | F | 2 |
| 9 | 128 | F | 2 |
| 10 | 138 | F | 4 |
| 11 | Cyan | O | 5 |
| 12 | Lime | O | 2 |
| 13 | Blue | O | 3 |
| 14 | Yellow | O | 6 |
| 15 | Turquoise | O | 4 |
| 16 | Sky | O | 5 |
| 17 | Violet | O | 4 |
| 18 | 48 | O | 5 |
| 19 | 78 | O | 3 |
| 20 | 118 | O | 2 |

*Bee species characteristics*

As described in the main text, three groups of bee species were included in the simulations: (i) specialists, (ii) generalists, and (iii) super-generalists. These differed in terms of their nest location (i.e. the starting point of the foraging journeys), their total flight distance and the total number of plant species that they could pollinate. Flight distance differed between groups by specifying different amounts of energy at the inception of model runs. The amount of energy used each time step was proportional to the step length (which was not fixed, but was drawn from a normal distribution). The flight was terminated when all energy was expended. All model simulations were continued until the flights of all bees had been completed (150 ticks). For the generalist and supergeneralist species, bee species were randomly allocated to plant species.

Table C. Characteristics of bee species. F = Forest, O = Open (non-forest) habitat. Note that the allocation of plant species to bee species was random (with the exception of bee species 12-20).

| Bee species identification number | No. of plant species can visit | Where nest | Distance flown when foraging | Which plant species can visit (plant species identification number). |
| --- | --- | --- | --- | --- |
| 1 (a) | 8 | F/O | 2000 | 3,5,8,10,11,14,16,18 |
| 2 (b) | 8 | F/O | 2000 | 4,5,9,13,14, 17,18,19 |
| 3 (c) | 8 | F/O | 2000 | 2,3,5,11,13,14,17,18 |
| 4 (d) | 4 | F/O | 1000 | 14,15,17,20 |
| 5 (e) | 4 | F/O | 1000 | 10,11,13,20 |
| 6 (f) | 4 | F/O | 1000 | 2,11,12,16 |
| 7 (g) | 4 | F/O | 1000 | 4,11,12,16 |
| 8 (h) | 4 | F/O | 1000 | 14,15,16,18 |
| 9 (i) | 4 | F/O | 1000 | 15,16,17,19 |
| 10 (j) | 4 | F/O | 1000 | 2,7,15,19 |
| 11 (k) | 4 | F/O | 1000 | 1,10,14,18 |
| 12 (l) | 1 | F | 500 | 2 |
| 13 (m) | 1 | F | 500 | 3 |
| 14 (n) | 1 | F | 500 | 4 |
| 15 (o) | 1 | F | 500 | 5 |
| 16 (p) | 1 | F | 500 | 6 |
| 17 (q) | 1 | F | 500 | 7 |
| 18 (r) | 1 | F | 500 | 8 |
| 19 (s) | 1 | F | 500 | 9 |
| 20 (t) | 1 | F | 500 | 10 |

*Calibration*

One issue that has received relatively little attention in the literature is how many pollinator visits are required to produce a robust network. Following Rivera-Hutinel et al. (2012), we evaluated the effect of sampling effort by producing species accumulation curves of the number of bee species included in the pollinator networks, by conducting model simulations of different durations (Fig. A). When the curve reaches an asymptote, this implies that the sampling effort is adequate to record the whole bee assemblage. The calibration curves were conducted without limiting the foraging distance of bee species. On the basis of the results obtained, a duration of 150 ticks was selected as the duration of all model runs.

Fig. A Species accumulation curves for model runs of different duration.

*Sensitivity analysis*

To assess sensitivity of model output to the allocation of bee species to plant species, three scenarios were conducted, using the same input maps for the deforestation gradient. Flower distributions and bee traits were the same as for the model runs described in the main text (see tables above). In the three scenarios, bee species were allocated randomly to flower species, as detailed on Table D. Only the listed combinations would lead to a flower visit being logged as a pollination event. In each of the scenarios, bee species 11-20 were randomly allocated to flower species 1-10, whereas bee species 1-10 were randomly allocated to flower species 1-20. Results are presented on Fig. B.

Table D. Allocation of bee species to plant species for the sensitivity analyses. Bee species 11-20 were randomly allocated to flower species 1-10, whereas bee species 1-10 were randomly allocated to flower species 1-20.

| Bee species identification number | Scenario 1.  Plant species identification numbers | Scenario 2  Plant species identification numbers | Scenario 3  Plant species identification numbers |
| --- | --- | --- | --- |
| 1 (a) | 1,3,4,8,9,14, 17,18, | 1,3,8,12,13,15,16,20 | 3,5,7,8,9,12,14,19 |
| 2 (b) | 3,7,8,9,11,14,16,19 | 2,4,5,10,12,13,14,20 | 1,2,4,10,12,13,15,17 |
| 3 (c) | 1,4,6,8,13,18,19,20 | 4,8,11,12,13,14,16,17 | 3,4,11,12,16,17,18,19 |
| 4 (d) | 1,4,6,13 | 2.3.4.6 | 4,7,10,13 |
| 5 (e) | 5,7,13,14 | 3,8,13,17 | 1,6,16,18 |
| 6 (f) | 1,13,18,19 | 2,9,14,15 | 6,13,15,19 |
| 7 (g) | 2,5,10,16 | 11,16,17,19 | 4,7,14,17 |
| 8 (h) | 1,3,12,19 | 2,6,13,16 | 4,6,11,12 |
| 9 (i) | 1,9,16,17 | 8,11,12,16 | 2,8,17,18 |
| 10 (j) | 7,10,11,12 | 10,13,16,19 | 1,4,13,16 |
| 11 (k) | 4,6,9,13 | 3,4,15,17 | 6,15,19,20 |
| 12 (l) | 1 | 6 | 7 |
| 13 (m) | 3 | 4 | 1 |
| 14 (n) | 7 | 5 | 1 |
| 15 (o) | 8 | 7 | 3 |
| 16 (p) | 6 | 6 | 1 |
| 17 (q) | 2 | 3 | 8 |
| 18 (r) | 4 | 8 | 5 |
| 19 (s) | 6 | 9 | 1 |
| 20 (t) | 1 | 10 | 2 |

Plant-pollinator networks are constructed from the interactions between plants and pollinators, and therefore the allocation of bee species to flower species is likely to have a major influence on the results. This was borne out by the results of the sensitivity analysis, which demonstrated significant variation between the three scenarios for each network metric (Fig. B).

Fig. B Sensitivity analysis. Filled circle, scenario 1; empty circle, scenario 2; filled triangle, scenario 3.

*Future development of the model*

In common with most of the literature on this topic, the current model simulated plant–pollinator interactions as the number of animal visits to flowers. However, the model could readily be amended to address pollen transfer or actual pollination events, for example by incorporating rules regarding the relationships between flower visits and pollination, variables such as the number, rate or duration of visits per plant, and the amount of pollen transported over different distances. Potentially ABMs could also help extend the bipartite networks that are typically employed for analysis of pollination networks to the individual level, offering the possibility of exploring ecological processes occurring at the scale of populations. The ABM presented here could also potentially be used to explore the temporal dynamics of network structure, which has been identified as a major knowledge gap.

In order to explain the impact of forest loss and fragmentation on pollinator networks more fully, there is a need to understand the mechanisms that might restrict pollinator species to larger forest fragments. This may include a requirement for specific nesting habitat or forest structure, as well as particular floral resources. Incorporation of such information could usefully form part of the calibration of the model to a specific field situation. The model could be further improved through inclusion of more accurate foraging behaviour. Here, no behavioural mechanism was included by which bees can locate or select foraging resources, and therefore the model does not assume that bees select foraging locations to maximize fitness. Furthermore, bee movement was simulated as a random walk, with no capacity for learning or trap-lining. Given that such processes have been observed in nature, pollination events could be predicted more accurately by incorporating bee movement according to foraging theory, as employed by some theoretical models (Olsson and Bolin 2014, Olsson et al. 2015). Further, the response of bees to visual and olfactory cues could also potentially be incorporated in future versions of the model, based on available experimental data.

**References**

Becher MA, Grimm V, Knapp J, Horn J, Twiston-Davies G, Osborne JL (2016) BEESCOUT: A model of bee scouting behaviour and a software tool for characterizing nectar/pollen landscapes for BEEHAVE. Ecological Modelling 340, 126-133.Boscolo D, Tokumoto PM, Ferreira PA, Ribeiro JW, Santos JS. (2017) Positive responses of flower visiting bees to landscape heterogeneity depend on functional connectivity levels. Perspectives in Ecology and Conservation 15 (1), 18–24.

Brosi BJ, Daily GC, Shih TM, Oviedo F, Durán G (2008) The effects of forest fragmentation on bee communities in tropical countryside. Journal of Applied Ecology 45 (3), 773–783.

Everaars J, Dormann CF (2014) Simulation of solitary (non-*Apis*) bees competing for pollen In: Devillers, J., (ed.) In silico bees. CRC Press, Boca Raton, FL, p. 209 – 268.

Ferreira PA, Boscolo D, Carvalheiro LG, Biesmeijer JC, Rocha PLB, Viana BF (2015) Responses of bees to habitat loss in fragmented landscapes of Brazilian Atlantic Rainforest. Landscape Ecology 30 (10), 2067-2078.Ferreira PA, Boscolo D, Lopes LE, Carvalheiro LG, Biesmeijer JC, da Rocha PLB, Viana BF (2018) . Forest fragmentation and connectivity loss simplify tropical pollination networks. Oecologia. In press.

Giannini TC, Garibaldi LA, Acosta AL, Silva JS, Maia KP, Saraiva AM, et al. (2015) Native and non-native supergeneralist bee species have different effects on plant-bee networks. PLoS ONE 10(9): e0137198. doi:10.1371/journal.pone.0137198

Lenz F, Chechkin AV, Klages R (2013) Constructing a stochastic model of bumblebee flights from experimental data. PLoS ONE 8(3): e59036. doi:10.1371/journal.pone.0059036

Marchand P, Harmon-Threatt AN, Chapela I (2015) Testing models of bee foraging behavior through the analysis of pollen loads and floral density data. Ecological Modelling 313*,* 41-49.

Michener, CD (2007) Bees of the World (2nd ed.) Johns Hopkins University Press, Baltimore, Maryland 953 pp

Olsson O, Bolin A (2014) A model for habitat selection and species distribution derived from central place foraging theory. Oecologia 175, 537–548.

Olsson O, Bolin A, Smith HG, Lonsdorf EV (2015) Modeling pollinating bee visitation rates in heterogeneous landscapes from foraging theory. Ecological Modelling [316](http://www.sciencedirect.com/science/journal/03043800/316/supp/C), 133–143.

O’Sullivan D and Perry GLW (2013) Spatial Simulation: Exploring Pattern and Process. Wiley, Chichester, England.

Ramalho AV, Gaglianone MA, de Oliveira ML (2009) Comunidades de abelhas Euglossina (Hymenoptera, Apidae) em Fragmentos de Mata Atlântica. Revista Brasileira de Entomologia 53(1), 95-101.

Reynolds AM (2009) Lévy flight patterns are predicted to be an emergent property of a bumblebees*’* foraging strategy. Behavioural Ecology and Sociobiology 64, 19–23.

Rivera-Hutinel A, Bustamante RO, Marín VH, Medel R (2012) Effects of sampling completeness on the structure of plant—pollinator networks. Ecology 93(7), 1593-1603.

Steffan-Dewenter I, Tscharntke T (1999) Effects of habitat isolation on pollinator communities and seed set. Oecologia 121 (3), 432–440.
