# Supplementary material for: Impacts of deforestation on plant-pollinator networks assessed using an agent based model
Source: PLoS One. 2018 Dec 31;13(12):e0209406. doi: 10.1371/journal.pone.0209406 (PMC6312366; doi:10.1371/journal.pone.0209406)
Supplement: S3 File — (DOC) [file pone.0209406.s003.doc]

# S3 Model description according to the ODD Protocol

The model description follows the ODD (Overview, Design concepts, Details) protocol for describing individual- and agent-based models (Grimm et al. 2006, 2010).

## 1. Purpose

## The purpose of the model is to understand the mechanisms influencing the dynamics and structure of plant-pollinator networks. The model was developed to provide a practical tool, which can be used to generate data of plant-pollinator interactions as a result of underlying mechanisms, including bee foraging and its interaction with the spatial distribution of flower resources, as influenced by the spatial characteristics of habitat pattern. Output of the model is designed to mimic the data obtained in field investigations, where visits by different pollinator species to individual plants are counted and used to construct plant-pollinator networks. Model output can be analysed in precisely the same way as conventional field data collected in this way.

***2. Entities, state variables and scales***

- *Agents/individuals*. Mobile agents are incorporated in the model, representing bees. Different bee species are indicated by different colours, and are incorporated in model code as different Netlogo breeds. Each bee individual is associated with an identity number, age, size, location (x y coordinates), energy reserves, metabolic rate (how rapidly they use energy at each time step), the amount of energy used at each timestep.
- *Spatial units (grid cells)*. Grid cells represent habitat type. In the model configuration presented here, the land cover is classified as either ‘Forest’ or ‘Open’ (non-forest) habitat. Maps of landcover generated by GIS or simulation tools such as GradientLand (Cambui et al. 2015) can be incorporated in the model to provide the distribution of grid cells of each habitat type. Some grid cells are then classified as flower resources, which can potentially be visited by bees. This is achieved by classifying the Netlogo grid cell as a particular colour, with different flower species represented by different colours. The model is configured such that when an individual bee visits an individual flower, this visit is logged; model output includes the sum of all visits made by each bee species to each flower species, summing across all individuals of each flower species.
- *Spatial and temporal scales and extents*. One time step represents approximately one minute and simulations were run for 150 minutes (150 ticks). One grid cell represents 10 x 10 m (= 0.01 ha) and the model landscape comprises 1,000 x 1,000 m; i.e., 1 square kilometer.

## 3. Process overview and scheduling

Time is modelled as discrete steps (ticks). At each timestep, the following processes occur:

*Move-bees.* The type of movement is selected from a pull-down menu on the model interface, and includes the following options: “random non-Levy”; “lattice”, “simple”, “normally distributed step length”, “Cauchy distributed step length”, “correlated directions”. These options are described in detail from the source from where this part of the model code was originally obtained from (O’Sullivan and Perry 2013). Once the type of movement has been selected, the agents move forward a distance that is determined by a slider (step-length) on the model interface. Once the movement has taken place, the amount of energy associated with each bee agent is reduced by the amount of energy used. At current configuration, the model employs “normally distributed step length”, and energy use is set to be equivalent to the step length.

*Count-visits.* Essentially the Netlogo grid cells that are classified as flowers count the number of visits by individuals of each bee species during that timestep. A visit is logged if the bee agent is located within the grid cell that is classified as a flower.

*Sum-visits*. This adds the number of visits of each bee species to each flower species to produce a running total. The duration of the simulation is set using a slider (duration) on the model interface, and the sum-visits procedure provides sums the total number of visits that occur within the set duration.

*Death*. This results in the death of any bee agent whose energy level has fallen to less than zero.

*Outputs*. Print output buttons on the model interface print out the total number of visits by all bee types to all flower types in a matrix, which is suitable for analysis of plant-pollinator networks (e.g. bipartite in R). This is output to a window on the interface screen, or alternatively using another button on the interface, the output can be saved to a text file.

## 4. Design concepts

*Basic principles.* The model is based on the assumption that bees forage randomly and encounter flowers by chance. In other words, there has been no attempt here to incorporate optimal foraging theory, or the sensing of flowers by olfactory or visual cues, although clearly such approaches could potentially be incorporated into future versions of the model. As currently configured, foraging is entirely random. Different functional types of bees (specialist, generalist, super-generalist) were configured within the model code, based on empirical observations (Gianni et al. 2015). These bee types differed in where they were initiated in the model (i.e. within Forest habitat, in the case of specialist species, or within either Forest or Open habitat, for the other two groups). Super-generalists and generalists were also provided with more energy (200 and 100) than specialists (50) at the outset of the simulation, with the effect that foraging flights were longer for super-generalists than generalists, and generalists than specialists. Again, this is based on empirical observations (e.g. see Gianni et al. 2015) rather than any underlying theory. The idea is that this model will provide insights into the mechanisms underlying variation in the structure of plant-pollinator networks, by enabling factors such as foraging behavior and its interaction with landscape pattern to be explored.

*Emergence*. The structure of the plant-pollinator network, as described by variables such as connectance, nestedness and network size, will emerge from the behavior of bee agents and their interaction with the spatial distribution of floral resources, and the pattern of habitat distribution at the landscape scale. We expect the different measures of plant-pollinator networks to vary in complex and perhaps unpredictable ways when the characteristics of bee-plant relationships are changed (eg the relative numbers of specialist versus generalist species), or in response to changes in the spatial distribution and pattern of habitat at the landscape scale.

*Adaptation*. None of the individuals display adaptive traits, as the model is currently configured.

*Objectives*. None of the agents have any specific objectives, other than to forage (in the case of bees).

*Learning.* The model does not incorporate any learning at present (although again this could potentially be incorporated in future iterations).

*Prediction*. The model does not incorporate any prediction by the agents.

*Sensing*. The only sensing that occurs does so at the scale of individual grid cells. Those classified as flowers will sense the presence of each bee species at each time step.

*Interaction*. There is no interaction between agents incorporated in the model, although some types of bee (i.e. specialists) are limited to nesting within particular habitats (i.e. forest). This ‘nesting behaviour’ simply refers to where the agents are located when the model is initiated.

*Stochasticity*. Movement of the bee agents is random or partly random, dependent on which of the movement procedures is selected. The location of both flowers and bee agents is random, but is governed by some additional rules (i.e. certain flower types are restricted to certain types of habitat, and certain bee types are located within particular habitat types when the model is initiated, as in the case of specialists located in forest habitat).

*Collectives*. Both the individual bee agents and the flowers belong to groups, namely the species. However these aggregations are used only in terms of reporting the total number of visits by each bee species to each flower species; the aggregations do not affect, and are not affected by, the individuals.

*Observation*. The output data that are captured are designed to precisely mimic what can be observed in an empirical study (following the “Virtual Ecologist” approach; Zurell et al., 2010). In other words, the number of visits by each bee species to each flower species is reported, in a format that can be directly analysed using tools conventionally used to analyse comparable field data (e.g. bipartite package in R).

## 5. Initialization

The initial state of the model world is partly determined by the values entered on the model interface, for a set of variables represented by sliders. These include the duration of the model simulation, the type of walk (i.e. movement of the bee agents), stdev-angle (employed on some of the options for type of walk), and mean step length. Model setup is effected by clicking on the ‘Setup’ button, which creates 20 flower species and 20 bee species, each of which has a different colour. At inception, bee agents are set with an energy level, metabolic rate, size, age and energy use, as well as random coordinates. Both bee agents and flowers are located randomly, although some species are restricted to particular land cover types, as determined in the model code. Setup also loads a raster file representing a map of land cover, as listed in the model code.

## 6. Input data

The model can incorporate raster files that represent a map of land cover, over which the bees forage. In the present configuration, a simple binary land cover classification is adopted (“Forest” versus “Open”). The raster files can be representations of real-world landscapes or virtual landscapes generated by tools such as GradientLand (Cambui et al. 2015).

## 7. Submodels

*Move-bees*. The formulae incorporated in the model are presented by O’Sullivan and Perry (2013).

*Count-visits*. The visits to individual flowers are summed.

*Sum-visits*. This sums all of the visits by each bee species to each flower species, combining data from all individuals of each species.

*Death*. This results in the death of any bee agent whose energy level has fallen to less than zero.

## 8. References

Cambui ECB, de Vasconcelos RN, Boscolo D, da Rocha PLB, Miranda JGV (2015) GradientLand Software: A landscape change gradient generator. Ecological Informatics 25, 57-62.

Giannini TC, Garibaldi LA, Acosta AL, Silva JS, Maia KP, Saraiva AM, et al. (2015) Native and non-native supergeneralist bee species have different effects on plant-bee networks. PLoS ONE 10(9), e0137198. doi:10.1371/journal.pone.0137198

Grimm V, Berger U, Bastiansen F, Eliassen S, Ginot V, Giske J, Goss-Custard J, Grand T, et al. (2006) A standard protocol for describing individual-based and agent-based models. Ecological Modelling 198, 115–126.

Grimm V, Berger U, DeAngelis DL, Polhill JG, Giske J, Railsback SF (2010) The ODD protocol: a review and first update. Ecological Modelling 221, 2760-2768.

O’Sullivan D and Perry GLW (2013) Spatial Simulation: Exploring Pattern and Process. Wiley, Chichester, England.

Zurell, D., Berger, U., Cabral, J.S., Jeltsch, F., Meynard, C.N., Münkemüller, T., Nehrbass, N., Pagel, J., Reineking, B., Schröder, B., Grimm, V., 2010. The virtual ecologist approach: simulating data and observers. Oikos 119, 22–635.
